# Community health worker motivation to perform systematic household contact tuberculosis investigation in a high burden metropolitan district in South Africa

**DOI:** 10.1186/s12913-020-05612-9

**Published:** 2020-09-18

**Authors:** Gladys Kigozi, Christo Heunis, Michelle Engelbrecht

**Affiliations:** Centre for Health Systems Research & Development, University of the Free State, Bloemfontein, South Africa

**Keywords:** Ward-based outreach teams, Free State Province, Community health worker, Primary health care

## Abstract

**Background:**

South Africa faces a chronic shortage of professional health workers. Accordingly, community health workers (CHWs) are being employed to mitigate the ongoing health workforce deficiencies. As increased access to quality service delivery hinges upon their motivation, this study explored CHWs’ motivation to deliver systematic household contact tuberculosis (TB) investigation (SHCI).

**Methods:**

In 2017, a cross-sectional survey was conducted among CHWs in the Mangaung Metropolitan District, Free State Province. Exploratory factor analysis was performed on a 30-item scale to determine the dimensions underlying CHW motivation. Items with factor loadings of 0.4 and above were retained. Descriptive and inferential analyses were used to determine CHW motivation levels. Multiple linear regression analysis was used to investigate the determinants of CHW motivation.

**Results:**

Out of 235 participants, 89.2% were female. Participants’ median age was 39 (inter-quartile range: 33–45) years. CHW motivation was defined by 16 items across three dimensions — intrinsic job satisfaction, burnout and team commitment, together explaining 56.04% of the total variance. The derived scale showed satisfactory internal consistency (Cronbach’s alpha: 0.81), with a mean motivation score of 52.26 (standard deviation [sd]: 5.86) out of 64. Statistically significant differences were observed between formal CHWs — those with at least phase 1 standardised accredited training, and informal CHWs — those without such accredited training regarding team commitment scores (17.82 [sd: 2.48] vs. 17.07 [sd: 2.82]; *t*_(233)_ = 2.157; *p* = 0.013). CHW age (β = 0.118, *p* = 0.029), location (β = 1.737, *p* = 0.041), length of service (β = − 0.495, *p* < 0.001), attendance of TB SHCI training (β = 1.809, *p* = 0.036), and TB SHCI competence (β = 0.706, *p* < 0.001), contributed statistically significantly to CHW motivation.

**Conclusion:**

CHW motivation to perform TB SHCI was both intrinsic and extrinsic. The high overall mean score implies that the CHWs were well-motivated to perform TB SHCI. To ensure sustained improved access to quality TB SHCI service provision, programme managers in the Free State and similar settings could potentially use the tool derived from this study to monitor and inform CHW motivation interventions. Interventions should pay close attention to the CHWs’ formalisation, competence and training.

## Background

In 2012, South Africa undertook to implement National Health Insurance (NHI) [1] as a means to achieve universal health coverage. A key health reform under the NHI is the re-engineering of primary health care (PHC) via three streams including municipal ward-based PHC outreach teams (WBPHCOTs), integrated school health programmes and district clinical specialist teams [2–4]. Organised under WBPHCOTs, community health workers (CHWs) constitute a critical human resource for the PHC re-engineering strategy. The CHWs are selected from the communities where they live and are accountable to the same communities [5, 6]. They are employed to mitigate the ongoing professional health workforce shortages in South Africa [2, 3]. In addition, they facilitate linkage between communities most in need and PHC facilities, thereby increasing access to basic health services and contributing to improved health [2, 3, 7–12].

At the time of this study in 2017, the implementation of WBPHCOT activities was largely guided by the *Provincial Guidelines on the Implementation of the Three Streams of Primary Health Care Re-engineering* released in 2011 [2, 4]. Subsequently, in 2018, a national *WBPHCOT Policy Framework* was launched to streamline the WBPHCOTs in terms of CHW selection, training, scope of work, monitoring and evaluation, and formal integration into the health system [2]. Ideally, a WBPHCOT comprises six to ten generalist CHWs, supervised by an outreach team leader (OTL) — usually an enrolled nurse who reports to the PHC facility manager — and a data capturer [2]. To be selected for the WBPHCOTs, individuals should preferably have a Grade 12 qualification. In terms of training, they typically receive initial short term training at recruitment to equip them with skills to provide safe and quality services, which is usually supplemented by refresher training to update their skills and reinforce the initial training [5, 7, 8, 13, 14]. A critique on the implementation of the WBPHCOT approach is that the all-important issues of CHW remuneration and working conditions are negated [3, 7]. Inevitably, poor remuneration and working conditions are bound to negatively affect CHWs’ motivation and performance, which in turn would affect their retention and the overall sustainability of the PHC re-engineering strategy.

Previous research has reported that both financial and non-financial incentives including, but not limited to, appreciation by communities, supportive supervision, acquisition of relevant skills, an opportunity for personal growth, the potential for career advancement, and feelings of altruism variably contribute towards CHW motivation [15–19]. Conversely, individual factors such as burnout, poor personal health and job insecurity, and environmental factors such as resource constraints (for example, lack of transport, medical supplies, etc.), inadequate remuneration, poor communication, and paucity of supervision and support, contribute towards CHW demotivation [17, 18, 20, 21].

There are few studies measuring motivation of CHWs in low- and middle-income settings such as South Africa. Moreover, the measurement of motivation in previous studies was not homogenous. Quantitative studies reported varied dimensions underlying CHW motivation [18, 19, 21], implying the necessity to contextualise the measurement of this construct. Therefore, this study contributes knowledge on important aspects of CHW motivation as measured quantitatively from a South African perspective.

In light of the ongoing PHC re-engineering in South Africa, this study focused on measuring motivation of CHWs recruited into WBPHCOTs in the Mangaung Metropolitan District in the Free State Province. Particularly, the study sought to measure and identify important aspects of CHW motivation to perform TB SHCI, as quality service delivery, and by extension, the success of the TB programme hinges upon their good motivation. Evidence across Africa indicates that there are substantial numbers of undiagnosed, untreated pulmonary tuberculosis (TB) cases in communities [22–25]. Consequently_*,*_ CHWs constitute a crucial component of the TB programme in South Africa. Their involvement in active case finding contributes towards the reduced transmission of TB in communities [25]. CHWs conduct systematic household contact TB investigation (SHCI) and refer undiagnosed individuals for clinical evaluation and treatment, trace patients lost to follow-up, and also provide basic behavioural and treatment adherence counselling [7, 9–11]. This study sought to understand the dimensions that underly CHWs’ motivation to perform SHCI, to ascertain the level of their motivation, and to establish the determinants of their motivation.

## Methods

### Design and setting

A cross-sectional study was conducted from October to December 2017 among CHWs in the Mangaung Metropolitan District, one of five districts constituting the Free State Province. In 2016, at 767 585, the metro had the second largest population of the five districts in the Free State and recorded sub-optimal TB outcomes in preceding years [26]. Mangaung Metropolitan was thus purposefully selected for the study. At the time of data collection, it constituted three sub-districts – Bloemfontein, Thaba Nchu and Botshabelo. However, the number of sub-districts has since increased to four.

### Participants

In 2016, there were 2 590 functional WBPHCOTs across South Africa [27] and 80 in the Free State Province [28]. Implementation of the WBPHCOT activities in the Province is ongoing and scale-up is largely dependent on the availability of funding. For this reason, the study participants comprised two categories of CHWs; informal CHWs — those who had not yet received standardised accredited WBPHCOT training, and formal CHWs — those who had received at least the first phase of the standardised accredited WBPHCOT training. By 2017, Mangaung Metropolitan had approximately 27 functional WBPHCOTs and 280 CHWs [29]. All CHWs enrolled into the WBPHCOTs in line with the extant provincial PHC re-engineering guidelines were purposefully selected for the study. The participants were recruited into the study through their OTLs. The latter were responsible for arranging appointments for fieldworker-administered interviews.

### Questionnaire development and data collection

A fieldworker-administered questionnaire was developed to gather data on CHW motivation, SHCI knowledge, competence and training, as well as socio-demographic characteristics (Additional file 1). Motivation refers to an individual’s willingness to exert and maintain effort towards organisational goals. It is the result of the interactions between individuals and their work environment, and the fit between these interactions and the broader societal context [30]. As motivation is a latent variable that cannot be easily assessed [30], items that measured various observable aspects of this variable were generated from existing literature on motivation among health workers, in particular studies conducted in low- and middle-income country settings [16–19, 31]. Constructs perceived to engender CHW motivation were considered, for example, general motivation, intrinsic motivation, burnout, job satisfaction, team commitment, conscientiousness, timeliness, altruism and compassion. At least three items were included for each construct. The questionnaire comprised a total of 30 items measuring CHWs’ motivation to perform TB SHCI on a Likert scale of 1 to 4 — 1 = strongly disagree, 2 = disagree, 3 = agree, and 4 = strongly agree.

Fourteen questions measured CHWs’ SHCI knowledge based on the national TB policy guidelines [32]. Participants were required to indicate ‘true’, ‘false’ or ‘don’t know’ in response to each question. Nine questions measured CHW TB SHCI competency based on the PHC re-engineering implementation guidelines [4] and national TB guidelines [32]. Participants had to indicate how difficult it was for them to perform certain TB SHCI tasks in households (i.e. 1 = difficult and 2 = not difficult). The questionnaire entailed a further ten questions measuring socio-demographic characteristics including sex, age, education, length of service, sub-district, CHW category (formal — with at least phase 1 of standardised accredited training vs. informal — without such accredited training) and TB SHCI training. The questionnaires were available in English and Sesotho. Trained bilingual fieldworkers administered the questionnaire via individual interviews conducted at the CHWs’ convenience and after obtaining informed consent. The interview duration was approximately 15 min.

### Data handling and analysis

The data were coded, double captured and verified for accuracy before analysis using SPSS version 25 [33]. Two data capturers individually entered the data into the programme. The data were then compared for any mismatches. Frequency distributions were also run on each variable to check for any errors and to ensure that data fell within the expected range.

In terms of analysis, CHWs’ socio-demographic characteristics were described using frequency counts and percentage for discrete variables, and mean, standard deviation (sd), and median for continuous variables. Exploratory factor analysis (EFA) was used to establish the dimensions underlying CHW motivation to perform TB SHCI [34]. The 30-item scale was analysed using EFA with principal axis factoring, oblique rotation and Eigenvalue greater than the Kaiser criterion of 1. Items with factor loadings of 0.4 and above and cross-loadings lower than 0.3 were retained. Cronbach’s alpha was used to examine the internal consistency of the derived motivational scale and sub-scales. Mean scores and t-tests were used to describe CHW motivation levels. Multiple linear regression analysis was used to establish the determinants of CHW motivation at a statistical significance level of *p* ≤ 0.05.

## Results

### Participant characteristics

In total, 235 CHWs participated in the study. A large majority were female (89.2%). The median age of the participants was 39 (inter-quartile range [IQR]: 33–45) years. The distribution of informal CHWs (49.4%) was similar to that of formal CHWs (50.6%). The majority (74.5%) of CHWs worked in small town areas. Regarding formal education, just more than three-quarters (75.7%) of the participants had attained a Matric/Grade 12 certificate. Most (61.3%) CHWs indicated that they were supervised by OTLs relative to PHC facility-based professional nurses/managers (28.1%) or non-governmental organisation (NGO)-based supervisors (9.8%). The median length of service was three (IQR: 1–6) years, the mean TB SHCI competence score was 7.1 (sd: 1.9) out of 9 and the mean TB SHCI knowledge score was 10.5 (sd: 1.2) out of 14 (Table 1).

**Table 1:** Participants’ demographic characteristics (*N* = 235)

Variable	n (%)
**Sex**	
Male	24 (10.2)
Female	211 (89.8)
**Age in years**: (median; IQR)	39 (33–45)
**CHW category**	
Informal	116 (49.4)
Formal	119 (50.6)
**Location**	
Urban	60 (25.5)
Small towns	175 (74.5)
**Formal education**	
Secondary school	42 (17.9)
Matric/Grade 12	178 (75.7)
Tertiary	15 (6.4)
**Supervisor**	
Outreach team leader	144 (61.3)
PHC facility-based nurse/manager	68 (28.9)
NGO-based supervisor	23 (9.8)
**Length of service in years** (median; IQR)	3 (1–6)
**Attended TB SHCI training in the past 12 months**	
Yes	164 (69.8)
No	71 (30.2)
**TB SHCI competency score** (mean out of 9; sd)	7.1 (1.9)
**TB SHCI knowledge score** (mean out of 14; sd)	10.5 (1.1)

*IQR* inter-quartile range, *sd* standard deviation

### Exploratory factor analysis of the motivation scale

The Kaiser-Meyer-Olkin measure verified sampling adequacy for the analysis (KMO = 0.838) and the Bartlett’s test of sphericity (X^2^[120] = 1469.639; *p* < 0.001) indicated that correlations between items were large enough for the EFA. Three factors had Eigenvalues over Kaiser’s criterion of 1 and in combination explained 56.04% of the variance. The scree plot showed inflexions that also justified retaining three factors. Given the convergence of the scree plot and Kaiser’s criterion on three factors, these three factors were retained in the final analysis. Table 2 shows the factor loadings after rotation. The clustering of the items on the factors suggested three distinct factors underlying CHW motivation. Factor 1 represented ‘intrinsic job satisfaction’; factor 2, ‘burnout’; and factor 3 ‘team commitment’. With an Eigenvalue of 5.30, factor 1 – ‘intrinsic job satisfaction’, comprised of six items and explained 33.14% of the variance in CHW motivation. Factor 2, − ‘burnout’, comprised of five items, had an Eigenvalue of 2.39 and explained 14.95% of the variance in CHW motivation. Factor 3 – ‘team commitment’ comprised of five items, had an Eigenvalue of 1.27 and explained 7.95% of the variance in CHW motivation. A total of 14 items were eliminated from the analysis because they did not contribute to a simple factor structure and failed to meet the minimum criteria of having a primary factor loading of 0.4 or above and no cross-loading of 0.3 or above. Subsequent analysis was based on the derived 16-item scale.

**Table 2:** Exploratory factor analysis of CHW motivation to perform SHCI

Item	Factor 1: Intrinsic job satisfaction	Factor 2: Burnout	Factor 3: Team commitment
I have happy thoughts and feelings about those I am able to help	**0.896**	0.006	−0.087
I am proud of what I can do to help others	**0.743**	0.000	−0.044
I believe I can make a difference in my work	**0.621**	−0.040	0.173
I am a hard worker	**0.574**	−0.034	0.170
I am punctual when coming to work	**0.492**	−0.032	0.109
I do things that need doing without being asked or told	**0.461**	0.035	0.217
I feel overwhelmed because my workload seems endless	−0.070	**0.793**^**a**^	0.160
I feel overwhelmed by my work as a CHW	−0.052	**0.673**^**a**^	0.212
Sometimes when I get up in the morning I dread having to face another day at work	0.047	**0.665**^**a**^	−0.119
I only do this job to get paid	−0.130	**0.448**^**a**^	−0.066
I feel emotionally drained at the end of every day	0.146	**0.416**^**a**^	−0.183
I am proud to be working for the WBPHCOT	0.051	−0.088	−**0.761**
I feel committed to working with this WBPHCOT	0.086	0.022	−**0.739**
The WBPHCOT inspires me to do my very best in my job working in the community	0.059	0.022	−**0.705**
I am satisfied with the services being provided by me	0.133	0.067	−**0.495**
My work makes me feel satisfied	0.235	−0.105	−**0.419**
**% variance explained**	33.14	14.95	7.95
**Cronbach’s alpha**	0.83	0.73	0.83
**Mean out of 4**	3.66	2.57	3.49
**Sd**	0.39	0.64	0.53

Extraction method: principal axis factoring. Rotation: Oblimin with Kaiser normalisation. ^a^Scores for negatively worded questions were reverse coded such that 1 = strongly agree, 2 = agree, 3 = disagree and 4 = strongly disagree, a high score suggested higher motivation; *sd* standard deviation

### Reliability analysis of the derived motivation scale

In terms of the reliability of the derived motivation scale as well as the associated individual sub-scales, internal consistency was calculated using Cronbach’s alpha. The 16-item scale showed satisfactory internal consistency with a Cronbach’s alpha of 0.81. Each of the individual sub-scales also had acceptable internal consistency as reflected by the respective Cronbach’s alpha values: intrinsic job satisfaction: 0.83; burnout: 0.73; and team commitment: 0.83.

### CHW motivation level

The mean motivation scores are depicted in Table 3. The scoring was such that higher mean scores suggested higher motivation. The highest mean score was established for the item “I am a hard worker” (mean: 3.78; sd: 0.45), implying that respondents would describe themselves as highly motivated in this respect. The lowest mean scores were for the item “I feel emotionally drained at the end of every day” (mean: 2.31; sd: 0.93) and “I feel overwhelmed by my work as a CHW” (mean: 2.31; sd: 0.95), implying that some respondents felt demotivated in these respects. The raw item scores were then summarised and the total score was divided by the total number of items measuring CHW motivation. The mean motivation score was 52.26 (sd: 5.86) out of 64 implying that overall, the CHWs were inclined to be well motivated to perform TB SHCI. In additional analysis, formal CHWs scored statistically significantly higher (*t*_(233)_ = 2.157; *p* = 0.013) than informal CHWs on the team commitment sub-scale (formal CHWs — mean: 17.82; sd: 2.48 vs. informal CHWs — mean: 17.07; sd: 2.82). However, job satisfaction and burnout scores were not significantly different across these groups. Comparison of motivation scores across the geographical areas yielded a statistically significant difference on the burnout scale, where CHWs from the urban area scored higher (*t*_(233)_ = 2.665; *p* = 0.008) than those from rural/small towns (urban area — mean: 13.10; sd: 3.13 vs. rural/small towns: — mean: 11.85; sd: 3.09).

**Table 3:** Level of motivation among CHWs (*N* = 235)

Item	n (%) agreed	Mean (sd out of 4)
**Job satisfaction**		
I have happy thoughts and feelings about those I am able to help	233 (99.1)	3.72 (0.47)
I am proud of what I can do to help others	234 (99.6)	3.76 (0.44)
I believe I can make a difference in my work	231 (98.3)	3.68 (0.50)
I am a hard worker	232 (98.7)	3.78 (0.45)
I am punctual when coming to work	222 (94.5)	3.52 (0.63)
I do things that need doing without being asked or told	222 (94.5)	3.52 (0.62)
**Burnout**		
I feel overwhelmed because my workload seems endless^**a**^	122 (51.9)	2.42 (0.94)
I feel overwhelmed by my work as a CHW^**a**^	136 (57.9)	2.31 (0.95)
Sometimes when I get up in the morning I dread having to face another day at work^**a**^	117 (49.8)	2.51 (0.97)
I only do this job to get paid^**a**^	28 (11.9)	3.29 (0.78)
I feel emotionally drained at the end of every day^**a**^	137 (58.3)	2.31 (0.93)
**Team commitment**		
I am proud to be working for the WBPHCOT	215 (91.5)	3.44 (0.78)
I feel committed to working with this WBPHCOT	217 (92.3)	3.47 (0.71)
The WBPHCOT inspires me to do my very best in my job working in the community	220 (93.6)	3.52 (0.64)
I am satisfied with the services being provided by me	222 (94.5)	3.50 (0.67)
My work makes me feel satisfied	220 (93.6)	3.52 (0.67)

Mean motivation score was 52.26 (sd: 5.86) out of 64; *N* = 235. ^a^Scores for negatively worded questions were reverse coded such that 1 = strongly agree, 2 = agree, 3 = disagree and 4 = strongly disagree, a high score suggested higher motivation; *sd* standard deviation)

### Determinants of CHW motivation to perform TB SHCI

Multiple linear regression analysis was performed to investigate whether CHW motivation to perform TB SHCI based upon CHWs’ sex, age, education, category, location, length of service, attendance of most recent TB SHCI training, and TB SHCI knowledge and competence. Preliminary analyses were performed to ensure that the assumptions of linearity, independence of errors, homoscedasticity, unusual points and normality of residuals were met. The results of the regression indicated that the model explained 16.4% of the variance and that the model was a significant predictor of CHW motivation to perform SHCI (F_9, 210_ = 4.109; *p* < 0.001; R^2^ = 0.164). Table 4 depicts the determinants of CHW motivation. CHW age (β = 0.118, *p* = 0.029), location (β = 1.759, *p* = 0.041), length of service (β = − 0.495, *p* < 0.001), attendance of TB SHCI training (β = 1.809, *p* = 0.036), and TB SHCI competence (β = 0.706, *p* < 0.001) contributed statistically significantly to motivation.

**Table 4:** Multiple regression model for the determinants of CHW motivation

Predictor	Unstandardised coefficient		*p*-value
	β	Standard error	
Constant	39.208	5.283	< 0.001
**Sex**			
Male (ref)	–		
Female	−1.578	1.216	0.196
**Age**	0.118	0.054	0.029
**Education**			
Secondary school (ref)	–		
Matric/Grade 12	−0.567	1.222	0.614
Tertiary	−2.310	1.874	0.219
**CHW category**			
Informal (ref)	–		
Formal	0.111	0.849	0.897
**CHW location**			
Urban (ref)	–		
Rural/small town	1.737	0.844	0.041
**CHW length of service**	−0.495	0.139	< 0.001
**Attended TB SHCI training in the past 12 months**			
No (ref)	–		
Yes	1.809	0.857	0.036
**TB SHCI knowledge score**	0.389	0.318	0.223
**TB SHCI competency score**	0.706	0.203	< 0.001

Overall *p* < 0.001; *R*^*2*^ = 0.164

## Discussion

South Africa subscribes to the World Health Organization’s (WHO) End TB Strategy envisioning to halt the TB epidemic by 2035 [35]. This ambitious goal necessitates commitment from TB programmes in terms of ensuring the quality of service provision. Moreover, quality service provision is largely dependent on health worker motivation [20, 21, 23, 25, 31, 36]. The study investigated the factors underlying the motivation of CHWs to perform TB SHCI in the Mangaung Metropolitan District of the Free State Province. Exploratory factor analysis established that CHW motivation was underscored by three dimensions including intrinsic job satisfaction, burnout and team commitment. The findings of this study corroborate previous research reporting that CHW motivation is both intrinsic and extrinsic [15, 18, 19, 21, 37–40].

The high mean scores suggest high overall CHW motivation to perform SHCI. More specifically, the high scores were in respect of the sub-scales measuring intrinsic job satisfaction and CHW commitment to the WBPHCOTs. The high motivation levels could potentially be attributed to social desirability bias among the CHWs, whereby they might have portrayed themselves in a better light—as being better motivated than they might have been [41]. Nevertheless, the 16-item scale used in this study showed satisfactory internal consistency with a Cronbach’s alpha of 0.81. This scale, in addition to other qualitative assessments, could be a useful tool for TB programme managers seeking to monitor and take actions to improve the CHW motivation levels in a bid to improve the quality of CHW service delivery.

In the current study, moderate levels of CHW burnout were evident. In a study that profiled CHWs in the KwaZulu-Natal Province of South Africa, burnout was attributed to sources external to the CHW including a lack of supportive supervision and career pathways, inadequate stipends, absence of debriefing following interactions with families, and dangerous work environments [40]. It is therefore imperative for TB programme managers/coordinators, WBPHCOT supervisors and policy makers to proactively improve CHWs’ working conditions in a bid to improve their motivation and retention. Enquiries into potential interventions deduced that multi-faceted interventions taking CHWs’ workloads and their expectations into account are likely to improve their motivation and retention in service for longer periods [42, 43].

While no significant differences were established across the CHW categories regarding job satisfaction and burnout, the current study found that formal CHWs scored significantly higher on the team commitment scale compared to informal CHWs. This is not surprising given that the latter group of CHWs – comprised almost 50% of the total number interviewed – were not formally recognised as being part of the healthcare workforce. The finding echoes calls from previous reports across South Africa to expedite the formalisation of CHWs in the health system [8, 39]. Maboko and colleagues [39] specifically urged that formal recognition of CHWs as an essential cadre for improving healthcare delivery at the community level, adequate remuneration for their work, as well as advanced training and a clear career development pathway, was needed to help to bolster their motivation and the quality of their services.

Results showed that CHWs who had attended TB SHCI training during the 12 months prior to the study scored higher on the motivation scale compared to those who had not. Likewise, motivation scores increased by 0.7 with every increasing unit of TB SHCI competence scores. These findings align with a previous study in the Free State Province reporting that more than half of the CHWs had not received basic training in human immunodeficiency virus counselling and testing [44]. In a Tanzanian study [45], the provision of regular refresher training was emphasised to ensure motivation and retention of CHWs in the country. It is therefore imperative for the TB programme in the Free State to provide the necessary initial and regular refresher training to CHWs. In three previous studies, one in the Eastern Cape Province, South Africa [46] and two in India [19, 21], CHWs expressed an urgent need for knowledge acquisition. The CHWs particularly felt empowered through the acquisition of knowledge and skills which facilitated their optimal functioning in communities [21]. Another study among CHWs in Uganda highlighted that skills and knowledge contributed to positive attitudes towards lay health work and positive intentions to remain in service [43]. However, while knowledge acquisition is crucial to the successful performance and individual level motivation of CHWs, under the PHC re-engineering strategy the current basic and continuing training systems remain largely fragmented and poorly organised [3].

The study further established that CHWs’ geographical location was a strong determinant of their motivation. CHWs in rural/small towns scored almost twice higher on the motivation scale compared to their counterparts in the urban area. This could potentially be associated with a greater sense of community connectedness that stems from the heavy reliance on CHWs for PHC in rural and remote areas [47]. Likely, the CHWs felt well recognised for their contribution to health care in the rural setting, leading to increased motivation to perform SHCI.

The strength of the study is that it contributes to knowledge on the levels of and dimensions underlying CHW motivation in a high TB-burdened metropolitan district. The findings from this study are important for TB policy and programme managers in the Mangaung Metro and other areas grappling with professional health workforce shortages amidst high disease burden. Moreover, this is the first study of CHW motivation to perform tasks related to the TB programme in the Metro, as well as the Province. However, the results of the exploratory factor analysis should be interpreted with caution. Future research in similar settings should validate the motivation scale. Moreover, only CHWs available on the day of the field visit were interviewed, which could likely explain the high levels of motivation [41]. Possibly, those who were absent were also less likely to be well motivated. Results are thus not generalizable to all CHWs in the province. Thus, future research should make provision to capture motivation levels of randomly selected CHWs through multiple field visits and qualitative assessments and also to explore the association between CHW motivation levels, turnover and the quality of services provided as this was not investigated in the current study. Another limitation is that the CHWs were only asked about their involvement in TB SHCI and this is only one of their many activities.

## Conclusion

This is the first study to measure the motivation of CHWs to perform TB SHCI in the context of PHC re-engineering in the Free State Province. CHW motivation was underscored by the dimensions of intrinsic job satisfaction, burnout and team commitment. The study found high levels of motivation among CHWs to perform TB SHCI. It is important to ensure sustained CHW motivation to effect improved access to quality service provision. The tool derived from this study could potentially be used by CHW supervisors in the Free State and similar settings to inform and monitor CHW motivation interventions. Interventions should especially pay close attention to the CHWs’ formalisation, competence and training.

## Supplementary information

**Additional file 1.** Survey questionnaire.

## Data Availability

The data analysed during this study are not publicly available as individual privacy would otherwise be compromised.

## Abbreviations

CHW: Community health worker
NGO: Non-governmental Organisation
NHI: National Health Insurance
OTL: Outreach team leader
PHC: Primary health care
SHCI: Systematic household contact investigation
TB: Tuberculosis
WBPHCOT: Ward-based primary health care outreach team
