# Supplementary material for: Community health worker motivation to perform systematic household contact tuberculosis investigation in a high burden metropolitan district in South Africa
Source: BMC Health Serv Res. 2020 Sep 18;20:882. doi: 10.1186/s12913-020-05612-9 (PMC7499870; doi:10.1186/s12913-020-05612-9)
Supplement: Supplementary file 1 — Additional file 1. Survey questionnaire. [file 12913_2020_5612_MOESM1_ESM.docx]

**Community Health Worker Questionnaire**

**Questionnaire number (office use only):**

**1. DEMOGRAPHIC INFORMATION**

1.1 Date today: dd _____________ mm _______________ yyyy ______________________

1.2 Sub-district: Bloemfontein Thaba Nchu Botshabelo

1.3 Name of fieldworker: __________________________________________________________

1.4 Note the sex of the respondent: Male Female

1.5 What was your age at your last birthday (years)? ________________________________________

1.6 What is the highest formal educational qualification that you have obtained?

No formal schooling

Primary school

Secondary school

Matric/Grade 12

Tertiary

1.7 What category of lay health worker are you?

Informal – Ward-based community care giver **Go to question 1.8**

Formal – Ward-based community health worker **Go to question 1.9**

1.8 How long have you been working as a ward-based community caregiver? **(If less than 1 year just specify number of months, if 1 year or more specify number of years)**

____________________ Years ____________________ Months **Go to question 1.10**

1.8.1 Who supervises your work?

__________________________________________________________________________________________

1.9 How long have you been working as a ward-based community health worker? **(If less than 1 year just specify number of months, if 1 year or more specify number of years)**

____________________ Years ____________________ Months

1.9.1 Who supervises your work?

__________________________________________________________________________________________

| 1.10 Since enrolling in your current job, have you received training relating to household contact TB investigation  Yes  No |
| --- |

**2. JOB-RELATED COMPETENCY**

**Indicate how difficult it is for you to perform the following tasks in households.**

| **Task** | **Difficult** | **Not difficult** |
| --- | --- | --- |
| 2.1 Conducting household risk assessments for TB |  |  |
| 2.2 Routinely screening household contacts of TB patients for TB |  |  |
| 2.3 Referring household contacts with possible TB to clinics for further assessment |  |  |
| 2.4 Recording household contacts with possible TB in case identification books |  |  |
| 2.5 Reporting those with possible TB to clinics |  |  |
| 2.6 Providing appropriate TB infection control information and education to people in households |  |  |
| 2.7 Encouraging household contacts with possible TB to attend clinics for further assessment |  |  |
| 2.8 Monitoring people on TB treatment for side effects |  |  |
| 2.9 Tracing TB treatment loss to follow up in households |  |  |

**3. KNOWLEDGE OF CONTACT INVESTIGATION**

**Mark with “X” the answer to each of the following statements.**

| **Statement** | **True** | **False** | **Don’t know** |
| --- | --- | --- | --- |
| - 1. A contact is a person who has shared airspace with an infectious TB patient |  |  |  |
| - 1. Close contacts of people with infectious TB are at increased risk of infection |  |  |  |
| - 1. Timely identification and adequate treatment of those with infectious TB reduces the risk of exposure to other people in the household |  |  |  |
| - 1. Household contact investigation means that **all** those living with patients with infectious TB should be screened for TB symptoms |  |  |  |
| - 1. All household contacts of MDR-TB patients should be screened for TB symptoms |  |  |  |
| - 1. Household contacts of HIV positive TB patients should be prioritised for TB investigation |  |  |  |
| - 1. Only children <5 years should be screened for TB in the household |  |  |  |
| **Statement** | **True** | **False** | **Don’t know** |
| - 1. Household contacts of patients with extra-pulmonary (outside the lungs) TB should be prioritised for TB investigation |  |  |  |
| - 1. Household contacts of patients with infectious TB should provide sputum for testing only if they are coughing |  |  |  |
| - 1. It is not necessary to assess households for risk factors for TB transmission once a patient has started TB treatment |  |  |  |
| - 1. Prolonged cough is a symptom of TB |  |  |  |
| - 1. Unintentional weight loss is a symptom of TB |  |  |  |
| - 1. TB can be spread through blood |  |  |  |
| - 1. Night sweats are a symptom of TB |  |  |  |

**4. LAY HEALTH WORKER MOTIVATION**

**To what extent do you agree with each of the following statements? Mark with “X” the appropriate response to each statement**.

| **Statement** | **Strongly Disagree** | **Disagree** | **Agree** | **Strongly Agree** |
| --- | --- | --- | --- | --- |
| 4.1 I feel motivated to work hard |  |  |  |  |
| 4.2 I only do this job to get paid |  |  |  |  |
| 4.3 I do this job because it gives security for me |  |  |  |  |
| 4.4 I feel emotionally drained at the end of every day |  |  |  |  |
| 4.5 Sometimes when I get up in the morning, I dread having to face another day at work |  |  |  |  |
| 4.6 I feel overwhelmed because of my work as lay health worker |  |  |  |  |
| 4.7 I feel overwhelmed because my workload seems endless |  |  |  |  |
| 4.8 Overall, I am very satisfied with my job |  |  |  |  |
| 4.9 I am not satisfied with my colleagues in my work |  |  |  |  |
| 4.10 I am satisfied with my supervisor |  |  |  |  |
| 4.11 I am satisfied with the health services being provided by me |  |  |  |  |
| 4.12 I feel that the services being provided by me are essential |  |  |  |  |
| 4.13 I get ample opportunities for career and skill development |  |  |  |  |
| 4.14 I am proud to be working for the ward-based outreach team |  |  |  |  |
| 4.15 I feel committed to working with this ward-based outreach team |  |  |  |  |
| 4.16 The ward-based outreach team inspires me to do my very best on my job working in the community |  |  |  |  |
| 4.17 I can rely on my colleagues at work |  |  |  |  |
| 4.18 I always complete my tasks efficiently and correctly |  |  |  |  |
| 4.19. I am a hard worker |  |  |  |  |
| 4.20 I do things that need doing without being asked or told |  |  |  |  |
| 4.21 I am punctual when coming to work |  |  |  |  |
| 4.22 I am often absent from work |  |  |  |  |
| 4.23 It is not a problem if I sometimes come late for work |  |  |  |  |
| 4.24 I get satisfaction from being able to help other people |  |  |  |  |
| 4.25 My work makes me feel satisfied |  |  |  |  |
| 4.26 I believe I can make a difference in my work |  |  |  |  |
| 4.27 I like my work as a lay health worker |  |  |  |  |
| 4.28 I have happy thoughts and feelings about those I am able to help |  |  |  |  |
| 4.29 I am proud of what I can do to help others |  |  |  |  |
| 4.30 I have thoughts that I am a success as a lay health worker |  |  |  |  |
